# Human-AI Collaboration for Remote Sighted Assistance: Perspectives from the LLM Era ^†^

**DOI:** 10.3390/fi16070254

**Published:** 2024-07-18

**Authors:** Rui Yu, Sooyeon Lee, Jingyi Xie, Syed Masum Billah, John M. Carroll

**Affiliations:** 1 Department of Computer Science and Engineering, University of Louisville, Louisville, KY 40208, USA; 2 Department of Informatics, Ying Wu College of Computing, New Jersey Institute of Technology, Newark, NJ 07102, USA; 3 College of Information Sciences and Technology, Pennsylvania State University, University Park, PA 16802, USA

**Keywords:** people with visual impairments, remote sighted assistance, conversational assistance, computer vision, artificial intelligence, human–AI collaboration, large language models

## Abstract

Remote sighted assistance (RSA) has emerged as a conversational technology aiding people with visual impairments (VI) through real-time video chat communication with sighted agents. We conducted a literature review and interviewed 12 RSA users to understand the technical and navigational challenges faced by both agents and users. The technical challenges were categorized into four groups: agents’ difficulties in orienting and localizing users, acquiring and interpreting users’ surroundings and obstacles, delivering information specific to user situations, and coping with poor network connections. We also presented 15 real-world navigational challenges, including 8 outdoor and 7 indoor scenarios. Given the spatial and visual nature of these challenges, we identified relevant computer vision problems that could potentially provide solutions. We then formulated 10 emerging problems that neither human agents nor computer vision can fully address alone. For each emerging problem, we discussed solutions grounded in human–AI collaboration. Additionally, with the advent of large language models (LLMs), we outlined how RSA can integrate with LLMs within a human–AI collaborative framework, envisioning the future of visual prosthetics.

## 1. Introduction

Remote sighted assistance (RSA) has emerged as a conversational assistive technology for people with visual impairments (PVI) [1]. In RSA paradigms, a user with a visual impairment uses their smartphone to establish a video connection with a remote sighted assistant, referred to as an *RSA agent* or simply an *agent*. The agent interprets the video feed from the user’s smartphone camera while conversing with the user to provide necessary or requested assistance. Recently, several RSA services have been developed both in academia, e.g., VizWiz [2], BeSpecular [3], and Crowdviz [4], and in the industry, e.g., TapTapSee [5], BeMyEyes [6], and Aira [7].

Historically, RSA services have been refined based on users’ needs and feedback from multiple trials [8-12]. Early RSA services featured unidirectional communication, i.e., from agents to users, with a narrow focus (e.g., agents describing objects in static images). As these services matured and gained popularity, both agents and users adopted new technologies such as smartphones, two-way audio/text conversation, real-time camera feed, GPS, and Google Maps. Consequently, current RSA services now support more complex tasks, such as assisting PVI in navigating airports and crossing noisy intersections without veering.

With the increased task complexity, researchers [1,13] have identified that reliance on smartphones’ camera feeds can be a limiting factor for agents, affecting their performance and mental workload, which can subsequently degrade the user experience of PVI. To elaborate, Kamikubo et al. [13] and Lee et al. [1], who studied RSA services, reported several challenges for agents, such as lack of confidence due to unfamiliarity with PVI’s physical surroundings, lack of detailed indoor maps, inability to continuously track PVI on static maps, difficulty in estimating objects’ distances in the camera feed, and describing relevant objects or obstacles in real time. However, these challenges are derived from the agents’ perspective, largely overlooking the user experience of PVI, who are the *true* users of RSA services. As such, the findings in prior works are likely to be incomplete. This paper draws on prior works to holistically understand the technical and navigational challenges in RSA from both the agents’ and users’ perspectives. Specifically, we aim to address two research questions: *What makes remote sighted assistance challenging?* and *When does this assistance become challenging to use?*

To that end, we employed two methodologies. *First,* we conducted a literature review to identify technical challenges in RSA services, mostly derived from agents’ point of view. *Second,* we conducted an interview study with 12 visually impaired RSA users to understand navigational challenges from their standpoint. Based on these two studies, we then constructed an exhaustive list of technical and navigational challenges to expand upon prior works and outline how these challenges occur in different real-world navigation scenarios. We organized technical challenges into four broad categories: agents’ difficulty in orienting and localizing the users, acquiring the users’ surrounding information and detecting obstacles, delivering information and understanding user-specific situations, and coping with poor network connection and external issues. Additionally, we produced a list of 15 real-world scenarios (8 outdoor, 7 indoor) that are challenging for PVI to navigate.

This article is an extended version of our work presented at the 2022 International Conference on Intelligent User Interfaces (IUI 2022) [14]. Compared with the conference paper, this extended article includes the following significant revisions:

First, in our conference paper, we initially explored how to use computer vision (CV) to address some identified challenges in RSA, but we mainly focused on how 3D maps can solve the first type of challenge (agents’ difficulty in orienting and localizing the users). In this extended article, we thoroughly analyze and outline the CV problems associated with each identified challenge, providing a detailed list of the challenges and corresponding CV problems. Additionally, we add a new section (Section 5), where we offer a detailed analysis of the relationships between each RSA challenge and CV problems. This new content is beneficial not only for HCI researchers considering how AI can improve RSA but also for CV researchers discovering new CV problems inspired by RSA.

Second, we analyze that some RSA challenges cannot be resolved with current CV technologies, opening up research opportunities for human–AI collaboration approaches. In our conference paper, we proposed five emerging problems in human–AI collaboration in RSA. Through a systematic review of CV problems in RSA, we now expand this list to 10 emerging problems: (1) making object detection and obstacle avoidance algorithms blind aware, (2) localizing users under poor network conditions, (3) recognizing digital content on displays, (4) recognizing text on irregular surfaces, (5) predicting the trajectories of out-of-frame pedestrians or objects, (6) expanding the field of view of live camera feeds, (7) stabilizing live camera feeds for task-specific needs, (8) reconstructing high-resolution live video feeds, (9) relighting and removing unwanted artifacts in live video, and (10) describing hierarchical information from live camera feeds. Section 6 provides a detailed discussion of each problem, supplemented with illustrative figures.

Third, the past two years have witnessed a revolution in AI technology, particularly with the rise of large language models (LLMs) [15] like GPT-4 [16], which have fundamentally changed how people perceive and utilize AI. In the realm of assisting PVI, the BeMyEyes [6] app has integrated GPT-4, introducing a BeMyAI [17] feature that delivers precise scene descriptions without needing remote sighted volunteers. In this transformative LLM era, we believe it is crucial to update our perspectives on human–AI collaboration in RSA. This extended article explores the potential of integrating RSA with LLMs and the new opportunities for human–AI collaboration, attempting to depict the future of visual prosthetics. These new insights are detailed in Section 7.

## 2. Background and Related Works

### 2.1. Navigational Aids for People with VI

Navigation is the ability to plan and execute a route to a desired destination. It is essential to have a spatial representation of users’ surroundings (i.e., digital maps, cognitive maps [18], building layouts), direction information, and continuous updates of their location in that representation (localization) [19]. Over the last 70 years, researchers have proposed many prototypes to aid PVI in both outdoor and indoor navigation. In this section, we only review a subset of such prototypes that are widely used and run on smartphones (for a chronological review, see Real and Araujo [20]).

Smartphone apps for outdoor navigation rely on GPS sensors for localization and commercial map services (e.g., Google Maps, OpenStreetMap [21]) for wayfinding. Examples include BlindSquare [22], SeeingEyeGPS [23], Soundscape [24], and Autour [25]. These apps are feasible for navigating large distances for PVI by providing spatial descriptions and turn-by-turn directions through audio. However, they are not reliable in the last few meters [26] due to a wide margin of error in GPS accuracy (±5m [27]).

The weaker GPS signal strength indoors is also a barrier to indoor navigation. To overcome this limitation, researchers have fused available smartphone sensors as alternatives for indoor navigation, such as motion sensors, Bluetooth [28], Infrared [29], NFC [30], RFID [31], sonar [32], beacon [33], and camera. The lack of sufficiently detailed indoor map data is the other challenge [34,35]. To mitigate this challenge, researchers have proposed constructing indoor maps by understanding the semantic features of the environment (for a complete list, see Elmannai and Elleithy [36]). Unfortunately, these solutions require additional deployment and maintenance effort to augment the physical environment [37], as well as a significant bootstrapping cost for setting up databases of floor plans [38] and structural landmarks [39,40]. Some solutions also require users to carry specialized devices (e.g., an IR tag reader [29]). For these reasons, no single indoor navigation system is widely deployed.

### 2.2. RSA Services for People with VI

RSA service is an emerging navigational aid for PVI [41]. The implementation of various RSA services differs in three key areas. (i) The communication medium between users and remote sighted assistants. Earlier prototypes used audio [8], images [2,12], one-way video using wearable digital cameras [9,42], or webcams [9], whereas recent ones use two-way video chat using smartphones [6,7,10,43]. (ii) The instruction form. RSA services are based on texts [44], synthetic speech [8], natural conversation [6,7,10], or vibrotactile feedback [11,45]). (iii) Localization techniques, for example, via GPS sensor, crowdsourcing images or videos [2,19,46,47], fusing sensors [19], or using CV as discussed in the next subsection.

Researchers have studied crowdsourced and paid RSA services. For crowdsourced RSA services (e.g., TapTapSee [5], BeMyEyes [6]), researchers concluded that they are feasible to tackle navigation challenges for PVI [48,49]. However, potential issues in crowdsourced RSA services include the following: (i) users trust too much in subjective information provided by crowdworkers, and (ii) crowdworkers are not available at times [50]. Compared with crowdsourced RSA services, Nguyen et al. [51] and Lee et al. [1] reported that assistants of paid RSA services (e.g., Aira [7]) are trained in communication terminology and etiquette, ensuring that they do not provide subjective information. Furthermore, they are always available.

### 2.3. Use of CV in Navigation for People with VI

Budrionis et al. [52] reported that CV-based navigation apps on smartphones are a cost-effective solution. Researchers have proposed several CV-based positioning and navigation systems by recognizing landmarks (e.g., storefronts [26]) or processing tags (e.g., barcodes [29,53], QR codes [54,55], color markers [56], and RFID [57]). CV techniques have also been applied to obstacle avoidance [58,59], which ensures that users can move safely during the navigation without running into objects. However, Saha et al. [26], who studied the last-few-meters wayfinding challenge for PVI, concluded that, for a deployable level of accuracy, using CV techniques alone is not sufficient yet.

Another line of work is to develop autonomous location-aware pedestrian navigation systems. These systems combine CV with specialized hardware (e.g., wearable CV device [60] and suitcase [61]) and support collision avoidance. While these systems have expanded opportunities to receive navigation and wayfinding information, their real-world adaptability is still questionable, as Banovic et al. [62] commented that navigation environments in the real world are dynamic and ever-changing.

Lately, researchers are exploring the feasibility of an augmented reality (AR) toolkit in indoor navigation, which is built into modern smartphones (e.g., ARKit [63] in iOS devices, ARCore [64] in Android devices). Yoon et al. [65] demonstrated the potential of constructing indoor 3D maps using ARKit and localizing users with VI on 3D maps with acceptable accuracy. Troncoso Aldas et al. [66] proposed an ARKit-based mobile application to help PVI recognize and localize objects. Researchers found that AR-based navigation systems have the advantage of (i) having widespread deployment [67], (ii) providing better user experience than traditional 2D maps [68], and (iii) freeing users’ hands without the need to point the camera towards an object or a sign for recognition [69].

More recently, we explored the opportunity of utilizing CV technologies to assist sighted agents instead of users with VI [70]. We designed several use scenarios and low-fidelity prototypes and had them reviewed by professional RSA agents. Our findings suggest that a CV-mediated RSA service can augment and extend agents’ vision in different dimensions, enabling them to see farther spatially and predictably and keeping them ahead of users to manage possible risks. This paper complements those findings by identifying situations where leveraging CV alone is not feasible to assist sighted assistants.

### 2.4. Collaboration between Human and AI

Despite recent advancements in CV, automatic scene understanding from video streams and 3D reconstruction remains challenging [71]. Factors such as motion blur, image resolution, noise, illumination variations, scale, and orientation impact the performance and accuracy of existing systems [71,72]. To overcome these challenges, researchers have proposed interactive, hybrid approaches that involve human–AI collaboration [73]. One representative of this approach is the human-in-the-loop framework. Branson et al. [74] incorporated human responses to increase the visual recognition accuracy. Meanwhile, they found that CV reduces the amount of human effort required. Similarly, researchers developed interactive 3D modeling in which humans draw simple outlines [75] or scribbles [76] to guide the process. They increased the accuracy of 3D reconstructions while considerably reducing human effort.

Collaborative 2D map construction and annotation is another example of human–AI collaboration, where AI integrates and verifies human inputs. Systems have been developed for collaborative outdoor (e.g., OpenStreetMap [21]) and indoor (e.g., CrowdInside [77], SAMS [78], and CrowdMap [79]) map construction. Researchers also investigated the use of collaborative 2D map construction and annotation in supporting navigational tasks for PVI, for example, improving public transit [80] and sidewalk [81,82] accessibility and providing rich information about intersection geometry [83]. Guy and Truong [83] indicated that collaborative annotations represent information requested by users with VI and compensate for information not available in current open databases.

Although prior works support the technological feasibility of collaborative mapping and annotation, the motivation and incentives of volunteers have been a concern surrounding collaborative map construction. Budhathoki and Haythornthwaite [84] indicated that volunteers can be motivated by intrinsic (e.g., self-efficacy and altruism) or extrinsic (e.g., monetary return and social relations) factors. In contrast, all volunteers are equally motivated in terms of a personal need for map data.

## 3. Identifying Challenges in RSA: Literature Review

We aimed to understand the challenges in RSA from two different perspectives, namely, the agents’ and users’ perspectives. This section presents a literature review that produces a list of such challenges from the agents’ perspective. Please refer to our IUI conference paper [14] for the detailed steps of our literature review methodology. The left part of Table 1 summarizes the identified challenges. Subsequently, we elaborate on the pertinent literature sources from which each individual challenge is derived.

### 3.1. Challenges in Localization and Orientation

One of the biggest challenges identified for RSA agents is accurately localizing users and orienting themselves. For this task, the agent mainly depends on the two sources of information—users’ live video feed and GPS location. The agents put them together to localize the users on a digital map on their dashboard [1,10]. However, the agents frequently get confused perceiving which direction the user is facing from the user’s camera feed and GPS location [9,13,42]. The trained agents who participated in a prior study [13] also reported that losing track of users’ current location is a challenging problem. RSA agents’ lack of environmental knowledge and their unfamiliarity with the area, the scarcity and limitations of the map, and the inaccuracy of GPS are found to be the main causes of location- and orientation-related challenges.

#### 3.1.1. Unfamiliarity with the Environment

In a previous study [101], the RSA agents expressed their frustrations with the users’ expectations of the agents’ quick start of assistance, which is usually not possible because most places are new to the agents, and thus, they need some time to process information to orient themselves. The fact that RSA agents have never been in the place physically and depend only on the limited map and the video feed is reported as a cause for the challenge in the research work [13,42,43].

#### 3.1.2. Scarcity and Limitation of Maps

Lee et al. [1] reported that RSA agents primarily use Google Maps for outdoor spaces, and they perform Google searches to find maps for indoor places. RSA agents who participated in Lee et al.’s study [1] reported that coarse or poor maps of malls or buildings limit their ability to assist users. They also stated that many public places either have no maps or have maps with insufficient details, which forces them to rely on another sighted individual in close proximity of the user for assistance. Sometimes, agents must orient users using their camera feeds only [1,42], which makes the challenges worse. Navigating complex indoor layouts is a well-established challenge in pedestrian navigation, as reported by many researchers [13,19,33,85].

#### 3.1.3. Inaccurate GPS

In addition to the insufficient map problem, inaccurate GPS was recognized as another major cause. Field trials of RSA systems [10] revealed that the largest orientation and localization errors occurred in the vicinity of a tall university building where GPS was inaccurate. Researchers [42] indicated that GPS signal reception was degraded or even blocked around tall buildings. In terms of the last-few-meters navigation [26], they illustrated that GPS was not accurate enough to determine whether the user was walking on the pavement or the adjacent road in some situations. The well-known last 10 meters and yard problem [26] in blind navigation is also caused by GPS inaccuracy.

### 3.2. Challenges in Obstacle and Surrounding Information Acquirement and Detection

The second notable challenge that agents face is obtaining information about obstacles and surroundings. RSA agents need to detect obstacles vertically at ground level to head height and horizontally along the body width [8,42,94]. They also need to provide information about dynamic obstacles (e.g., moving cars and pedestrians) and stationary ones (e.g., parked cars and tree branches) [10,94]. However, agents found these tasks daunting due to the difficulties in estimating the distance and depth [13], reading signages and texts [43], and detecting/tracking moving objects [43,94] from the users’ camera feed. A number of research studies have also found that it is almost impossible for agents to project or estimate out-of-frame potential obstacles, whether moving or static [8-11,13,42,94]. Researchers [62] describe that navigation environments in the real world are dynamic and ever-changing. Thus, it is easier for agents to detect obstacles and provide details when users are stationary or moving slowly [43]. Two main causes are linked with the aforementioned problems: (1) limited field of view (FOV) of the camera and (2) limitation of using video feed.

#### 3.2.1. Narrow View of the Camera

Prior research found that the camera in use had a relatively limited viewing angle of around 50°, compared with the angle of human vision, which is up to 180° [9]. Researchers [11,94] mentioned that the camera should be located appropriately to maximize vision stability along the path. A limited field of camera view affects RSA agents negatively in their guiding performance [13,108].

#### 3.2.2. Limitation of Using Video Feed

The quality of the video feed that matters to the RSA is the steadiness and clearness. The video stream is easily affected by the motion of the camera (e.g., handheld mobile device or glasses) and becomes unstable. It is reported that agents are more likely to experience motion sickness when users are not holding the camera (e.g., smartphone hanging around the user’s neck) [43]. To mitigate the challenges of reading signages and texts in the user’s camera feed, researchers [9] demonstrated the necessity of enhancing the quality of the video stream. A smooth frame rate and high resolution are essential when agents read signs, numbers, or names. The quality of the video stream can affect the performance of RSA in hazard recognition [109,110], and thus, it is considered as one of the main factors determining the safety of blind users [10]. This explains why the task of an intersection crossing was recognized as one of the most challenging situations for agents in a navigation aid. RSA agents find it very challenging because it is difficult to identify traffic flow through the narrow camera view, poor video quality, and the high speed of vehicles [9,13,43].

### 3.3. Challenges in Delivering Information and Interacting with Users

In addition to the challenges in the task of obtaining the necessary information, agents report the next set of challenges occurring in delivering the obtained information and interacting with users. In previous studies [1,101], agents revealed the difficulties in providing various pieces required of information (e.g., direction, obstacle, and surroundings) in a timely manner and prioritizing them, which requires understanding and communicating with users, which creates further challenges. The agents could also become stressed if users move faster than they could describe the environment [1]. These suggest that, in the navigation task, the need to deliver a large volume of information and, simultaneously, the need to quickly understand each user’s different situation, need, and preference are the main causes for the challenges. Prior research found that RSA agents deal with these challenges through collaborative interaction/communication with users [1,43].

### 3.4. Network and External Issues

Early implementations of RSA services suffered from the network connection and limited cellular bandwidth [42]. Although cellular connection has improved over the years, the problem remains for indoor navigation [43], which could lead to large delays or breakdowns of video transmissions [13,94,111]. Additionally, an external factor such as low ambient light conditions at night causes the poor quality of the video feed.

## 4. Identifying Challenges in RSA: User Interview Study

Next, we conducted a semi-structured interview study with 12 visually impaired RSA users to understand the challenges from the perspective of RSA users’ experience. In this section, we report the findings of the users’ experienced challenges, their perceptions of RSA agents’ challenges, and how the challenges on each side of the RSA provider and users are related and affect the RSA experience. Please refer to our IUI conference paper [14] for specific information regarding participants, procedures, data analysis, and other details of this user study.

From the interview study, we identified challenging indoor and outdoor navigational scenarios from the blind users’ experience (Table 2). Further, we saw that major problems recognized from the literature review (e.g., the limitations of maps, RSA’s environmental knowledge, and the camera view and feed) reappear as the main causes for challenges for blind users and found how those problems affect users’ navigation experience and how they perceive and help address the problems on the users’ end.

### 4.1. Common Navigation Scenarios

The most common types of navigation scenarios that our participants asked RSA agents for help with are traveling and navigating unfamiliar indoor or outdoor places. Navigating unfamiliar areas where a blind user might utilize the RSA service came up often in our study, which is consistent with the literature [1,101]. For outdoor navigation, common scenarios include checking and confirming the location after Uber or Lyft drop-offs; finding an entrance from a parking lot; taking a walk to a park, coffee shop, or mailbox; navigating in a big college campus; and crossing the street.

The common indoor places they called RSA agents for help with were airports and large buildings (e.g., malls, hotels, grocery stores, and theaters). In an airport, they usually asked RSA agents to find a gate and baggage claim area. Inside large establishments or buildings, they asked for finding a certain point of interest (e.g., shops, customer service desk); entrances and exits, stairs, escalator, and elevator; and objects, e.g., vending machines and trash cans. Our data suggest that blind users repeatedly use RSA services to navigate the same place if its layout is complex (e.g., airports), or their destination within the place is different (e.g., different stores in a shopping mall), or the place is crowded and busy (e.g., restaurants).

### 4.2. Challenging Outdoor Navigation Experiences

It was a recurrent theme that if the agents experienced challenges, the users also experienced challenges. The interviewees were mostly content with their outdoor navigation experience with the agent, compared with that in indoor navigation, even though they realized that some scenarios were challenging to agents. Examples of such scenarios include crossing intersections and finding certain places and locations (e.g., building entrances, and restrooms) in open outdoor spaces (e.g., parking lots, campuses).

### 4.3. Challenging Indoor Navigation Experiences

All our interviewees commonly mentioned that indoor navigation was more challenging for them, as well as for the agents. Interviewees’ indoor experiences with RSA indicate that it usually took much longer for the agent to locate and find targets in indoor spaces. Christi shared her challenging experience of spending about 20 minutes with an RSA agent only to find a store (“Bath and Body Works”) from another store in a big mall. Another interviewee, Rachel, recounted the longest and the most challenging time she had with an agent when trying to find the luggage section in a department store, Kohl’s.

Finding the entrance (or exit) of a building and navigating to a pick-up point from the interior of a building to meet the ride-sharing driver are examples of other challenging experiences that our interviewees shared with the RSA agents.

### 4.4. Users’ Understanding of Problems: Insufficient Maps, RSA’s Unfamiliarity of Area, and Limited Camera View

All interviewees mentioned that the absence of maps or floor plans and the inaccuracy and scarcity of the map were the primary reasons why RSA agents struggled to assist them in both indoor and outdoor places. Most interviewees also mentioned that the RSA agent’s unfamiliarity with a place and its location is a significant challenge.

Several interviewees’ accounts also suggested that poor and limited visibility caused by the narrow camera view creates challenges for the agents. Karen talked about using stairs in her school and showed her understanding of the agent’s visual challenge caused by the limited distance that the camera feed can show. She also introduced another visual challenge that the current camera’s capability cannot solve, and Calvin’s comment implies the same issue.

### 4.5. Users Helping and Collaborating with RSA

The participants seem to understand the difficulties and situations that the agents face on their end, and they want to assist the agents and are willing to work with them to mitigate the challenges and complete the navigation task together. Karen and Grace shared what they usually did to help the agent get a better view of the video feed for the direction and distance on their end. Karen said that she paid careful attention to positioning the phone correctly so that the agent could see what they needed to see. The participants also mentioned that a common workaround that RSA agents use in challenging scenarios is to find a dependable sighted person, such as an employee with a uniform or someone at the help desk, who could help them quickly. Lee et al. [1] also reported a similar finding.

## 5. Identified Computer Vision Problems in RSA

Since the identified challenges are mostly related to visual understanding and spatial perception, we relate one or more CV problems to each challenge in Table 1. The related CV problems include object detection, visual SLAM, camera localization, landmark recognition, visual navigation, scene text recognition, depth estimation, scale estimation, multiple object tracking, human trajectory prediction, field-of-view extrapolation, video stabilization, visual search, video captioning, video super-resolution, and video relighting. These problems are well formulated in the CV literature. Researchers have developed various methods to address these problems and have mostly evaluated them on standard benchmark datasets. In this section, we will analyze the applicability of each of these CV problem settings and solutions when dealing with the identified RSA challenges so as to find out the unique requirements of RSA services for CV research.

### Object detection [102]

aims to detect instances of objects of predefined categories (e.g., humans, cars) in images or videos. Object detection techniques can answer a basic CV question: what objects exist in the image and where are they? It can be used to help address the RSA challenge of difficulty in proving various information, i.e., problem G3.(1) in Table 1. For example, if the user wants to find a trash can, the object detection algorithm can continuously detect objects in the live video feed. When a trash can is detected, it will notify the agent and user.

### Visual SLAM [86]

methods are used for mapping an unknown environment and simultaneously localizing the camera through the video captured in this environment. It can directly address the RSA challenges of scarcity of indoor maps and inability to localize the user, i.e., problem G1.(1)(2)(3) in Table 1. The benefit of visual SLAM for RSA services has been demonstrated in our prior work [112] using Apple’s ARKit.

### Camera localization [87]

aims to estimate the 6-DOF camera pose (position and orientation) in a known environment. Here, the camera is a general term and could include an RGB camera, RGB-D camera, or 3D LiDAR. Camera localization can address the RSA challenges of inability to localize the user and difficulty in orienting the user, i.e., problem G1.(2)(3) in Table 1. Similar to visual SLAM, our prior work [112] has shown that real-time camera localization can effectively support RSA in indoor navigation.

### Landmark recognition [88]

finds the images of a landmark from a database given a query image of the landmark. It is a subtask of content-based image retrieval and has the potential to address the RSA challenges of lack of landmarks on the map, i.e., problem G1.(4)(5) in Table 1. Instead of annotating landmark locations on the map, we can crawl and save the pictures of the landmark in a database. Then, when the user explores the environment, the landmark recognition algorithm can search the database and automatically annotate the landmark on the map if it matches. However, it may be only valid for well-known landmarks. For subtle landmarks, the challenge is open for exploration.

### Visual navigation [89]

requires a robot to move, locate itself, and plan paths based on the camera observations until reaching the destination. It may help address the RSA challenges of last-few-meters navigation [26], i.e., problem G1.(7) in Table 1.

### Scene text recognition [90]

aims to recognize text in wild scenes from a camera and can be regarded as camera-based optical character recognition (OCR). It is still a challenging CV problem due to factors such as poor imaging conditions. The scene text recognition technique could be used to overcome the RSA challenge of difficulty in reading signages and texts from the camera feed, i.e., problem G2.(1) in Table 1.

### Depth estimation

is a task of estimating the depth of a scene. In particular, monocular depth estimation [91] aims to estimate depth from a single image. It could be used to address the RSA challenge of difficulty in estimating the depth from the user’s camera feed and in conveying distance information, i.e., problem G2.(2) in Table 1.

### Scale estimation [92,93,113]

not only determines the distance between the camera and an object (e.g., an obstacle) but also provides an estimate of the object’s real size, leading to a more accurate understanding of the environment. However, due to the inherent problem of scale ambiguity in monocular SLAM, it is often necessary to combine the camera with other sensors, such as an inertial measurement unit (IMU) or depth sensor, or use multiple cameras to achieve precise scale estimation with a smartphone. Once the 3D environment with scale information is reconstructed, it can be leveraged to better address the RSA challenge of difficulty in estimating the depth from the user’s camera feed and in conveying distance information, i.e., problem G2.(2) in Table 1.

### Multiple object tracking [95]

aims to identify and track objects of certain categories (e.g., pedestrians, vehicles) in videos. It is the basis of downstream tasks such as human trajectory prediction and could help address the RSA challenge of difficulty in detecting and tracking moving objects, i.e., problem G2.(3) in Table 1.

### Human trajectory prediction [96,97,114]

aims to understand human motions and forecast future trajectories considering interactions between humans and constraints of the environment. It could be used to solve the RSA challenge of difficulty in tracking moving objects and inability to project or estimate out-of-frame people from the camera feed, i.e., problem G2.(3)(4) in Table 1.

### Field-of-view extrapolation [98,99]

is a task of generating larger FOV images from small FOV images using the information from relevant video sequences. It has the potential to address the RSA challenge of inability to estimate out-of-frame objects or obstacles from the user’s camera feed, i.e., problem G2.(4) in Table 1.

### Video stabilization [100]

can remove the serious jitters in videos captured by handheld cameras and make the videos look pleasantly stable. Video stabilization techniques can reduce the RSA challenge of motion sickness due to an unstable camera feed, i.e., problem G2.(5) in Table 1.

### Visual search

deals with the problem of searching images with the same content as the query image from a database. In particular, person search [103,104,115-117] aims to find the same person as the query image from a database of scene images. It needs to identify both which image and where on the image, and has the potential to address the RSA challenge of difficulty in proving various information, i.e., problem G3.(1) in Table 1. Similar to object detection, person search algorithms can continuously detect people on the live video feed. When the specific person is found, it will alert the agent and user.

### Video captioning [105]

is a task of describing the content of videos with natural language, which combines CV and natural language processing (NLP). It might help address the RSA challenge of adjusting the level of detail in description provision through communication, i.e., problem G3.(2) in Table 1. When the RSA agent is processing information (e.g., reading the map) or misses some information in the description, video captioning techniques can help describe the environment for the user.

### Video super-resolution [106]

is a classic CV and image processing problem, aiming to recover a video from low resolution to high resolution. It can be used to reduce the RSA challenge of poor quality of the video feed, i.e., problem G4.(2) in Table 1.

### Video relighting [107]

aims to recalibrate the illumination settings of a captured video. It may also help address the RSA challenge of poor quality of the video feed, i.e., problem G4.(2) in Table 1, when the ambient light condition is unsatisfactory (e.g., at night).

## 6. Emerging Human–AI Collaboration Problems in RSA

While examining RSA challenges, we identified associated CV problems for each challenge. However, existing CV research has been mostly conducted using specific problem formulation and benchmark datasets, which are not conducive to the unique application scenarios of RSA. RSA involves handheld mobile camera equipment with a narrow FOV, where the holder (PVI) cannot see the surrounding environment or the screen. Consequently, deploying models trained on benchmark datasets directly onto PVI’s mobile phones leads to a significant drop in accuracy due to domain gaps. Thus, most existing CV models are not directly applicable to addressing RSA challenges.

Nevertheless, it is important to recognize that, in RSA, the remote assistant is a sighted individual. Leveraging the assistance of sighted agents could reduce the strict requirements on CV models. Consequently, adopting a human–AI collaboration approach becomes crucial in mitigating RSA challenges. For those RSA challenges beyond the scope of existing CV techniques, we identified 10 emerging human–AI collaboration problems. We also analyzed potential solutions based on human–AI collaboration as follows.

### 6.1. Emerging Problem 1: Making Object Detection and Obstacle Avoidance Algorithms Blind Aware

Obstacle avoidance is a major task in PVI’s navigation due to safety concerns. As discussed in the literature review, detecting obstacles is a notable challenge through the narrow camera view, because the obstacle could appear vertically from ground level to head height and horizontally along the body width [8,42,94], as listed in problem G3.(1) in Table 1. This requires agents to observe obstacles at a distance from the camera feed. However, it is still extremely difficult for agents because the obstacles afar would be too small to recognize in the camera feed. The challenge motivates us to resort to AI-based object detection algorithms [118], which are able to detect small objects. However, it is problematic to directly apply existing object detection algorithms [119,120] to the RSA services. For example, a wall boarding a sidewalk is considered as an obstacle in common recognition models but can be regarded as orientation and mobility (O&M) affordances for people with VI who use a cane and employ the wall as a physical reference. We term the ability of recognizing affordances that are important for people with VI as *blind aware*, a common philosophy in end-user development [121]. Due to the importance of detecting obstacles in a blind-aware manner, we consider it as an emerging research problem that can be addressed by human–AI collaboration.

In the context of navigation, studies have adopted machine learning algorithms to automatically detect and assess pedestrian infrastructure using online map imagery (e.g., satellite photos [122,123], streetscape panoramas [124-126]). A recent work [127] applied ResNet [128] to detect accessibility features (e.g., missing curb ramps, surface problems, sidewalk obstructions) by annotating a dataset of 58,034 images from Google Street View (GSV) panoramas.

We can extend these lines of work to a broader research problem of detecting objects including accessibility cues in navigation. First, we need volunteers to collect relevant data from satellite photos (e.g., Google Street, open-street maps), panoramic streetscape imagery, 3D point clouds, and camera feeds of users. Following [127], data-driven deep learning models are trained with human annotated data. It is worth noting that the data are not limited to images but also 3D mesh or point clouds, especially considering that iPhone Pro is equipped with a LiDAR scanner. To train blind-aware models for object detection, we also need to manually define whether an object is blind aware with the help of PVI. Specifically, blind users can provide feedback on the quality of a physical cue [129]. Additionally, another human–AI collaboration direction is to online update the CV (e.g., obstacle detection) models with new navigation data marked by the agents. Solving this problem could make blind navigation more customized to how the blind user navigates through space and expedite the development of the automated navigation guidance system for blind users.

### 6.2. Emerging Problem 2: Localizing Users under Poor Networks

Although cellular bandwidth has increased over the years, the bad cellular connection is still a major problem in RSA services, especially in indoor navigation [43], as listed in problem G4.(1) in Table 1. The common consequences include large delays or breakdowns of video transmissions [13,94,111]. Suppose that the poor network only allows transmitting limited amount of data and cannot support live camera feed, it is almost impossible for agents to localize the user and give correct navigational instructions. Based on this observation, we identify an emerging research problem of localizing users under poor networks that can be addressed by human–AI collaboration.

**Figure 1.**
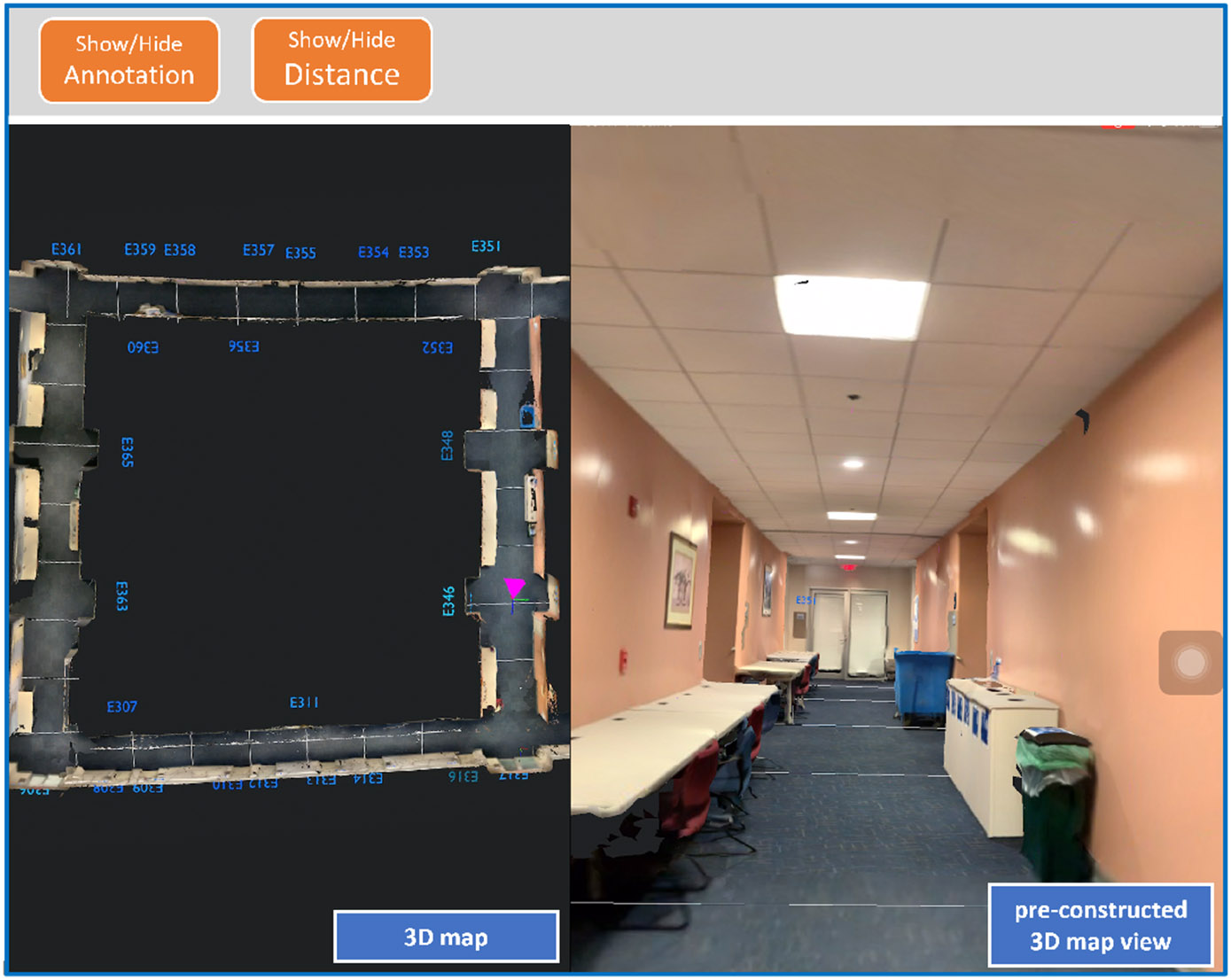
Our design prototype for localizing users under poor networks with a split-screen dashboard. The top toolbar shows buttons to toggle a design feature on or off. The left-side screen shows a top–down view of a pre-constructed indoor 3D map, with the pink shape representing the user’s location and orientation. The right-side screen shows a pre-constructed 3D map view, which supplements the live camera feed under poor networks.

With regard to AI–based methods, one possible solution is to use interactive 3D maps, constructed with ARKit [63] using an iPhone with a LiDAR scanner, as shown in Figure 1. During an RSA session under a poor network, the user’s camera can relocalize them in the 3D maps. If their location and camera pose is transmitted to agents, agents can simulate their surroundings on the preloaded offline 3D maps. Considering the camera pose can be represented by a 4 × 4 homogeneous matrix, the transmitted data size is negligible. With voice chat and the camera pose displayed on the 3D maps (e.g., top–down and egocentric views in Figure 1), the agent can learn enough information about the user’s surroundings and localize the user under a poor network momentarily.

In terms of human–AI collaboration, to the best of our knowledge, there is no work for RSA on localizing users under poor networks. Without live camera feed, it would be a more interesting human–AI collaboration problem. To localize the user in such a situation, the communication between the agent and the user would be greatly different. We can imagine some basic communication patterns. First, the agent can ask the user to make certain motions (e.g., turn right, go forward) to verify the correctness of the camera pose display. In turn, the user can actively ask the agent to confirm the existence of an O&M cue (e.g., a wall) from the 3D maps. It is worth noting that the offline 3D map could be different from the user’s current surroundings. When exploring the map, they also need to work together to eliminate the distraction of dynamic objects (e.g., moving obstacles), which do not exist on the 3D map. The specific problems have never been studied in detail, for example, how to detect localization errors and maintain effective RSA services with low data transmission rates.

### 6.3. Emerging Problem 3: Recognizing Digital Content on Digital Displays

Digital displays, such as LCD screens and signages, are widely used in everyday life to present important information, e.g., flight information display boards at the airport, digital signage at theaters, and temperature control panels in the hotel. RSA agents reported difficulty in reading texts on these screens when streamed through the users’ camera feed, as listed in problem G2.(1) in Table 1. This difficulty can be caused by several technical factors, including the varying brightness of a screen (i.e., the display of a screen is a mixture of several light sources, e.g., LCD backlight, sunlight, and lamplight [130]); a mismatch in the camera’s frame rate and the screen’s refresh rate; and a mismatch in the dimension of pixel grids of the camera and the screen, resulting in moire patterns, i.e., showing strobe or striping optical effects [130]. Based on the significance and challenges of recognizing content on digital displays through camera feeds, we consider it as an emerging research problem that can be addressed by human–AI collaboration.

From the perspective of AI solutions, there exist a few CV systems that assist blind users in reading the LCD panels on appliances [131-134]. However, these systems are heuristic driven and fairly brittle and only work in limited circumstances. To the best of our knowledge, there is no text recognition method specifically designed to recognize digital texts on LCD screens or signages in the wild.

In this regard, we consider scene text detection and recognition [135] as the closest CV method aiming to read texts in the wild. However, these methods are far more difficult than the traditional OCR of texts from documents. For example, the state-of-the-art deep learning methods [136-138] only achieve < 85% recognition accuracy on the benchmark dataset ICDAR 2015 (IC15) [139]. Furthermore, existing methods for scene text recognition are likely to suffer from the domain shift problem due to the distinct lighting condition [140], resulting in even worse recognition performance in reading digital content on LCD screens.

To formulate human–AI collaboration, we consider scene text recognition methods [135] as the basis for AI models. Next, we consider three aspects of human–AI collaboration. First, CV techniques can be used to enhance the camera feed display [141], while the agents are responsible for the content recognition. In this way, the content in the live camera feed will be transferred to have better lighting and contrast, making it more suitable for the agents to perceive and recognize.

Second, scene text recognition methods [135] can be used to read digital content for the agents and provide recognition confidence. This approach is particularly useful for recognizing small-scale text that is too small for agents to read on the camera display but contains enough pixels for AI models to process. The agent can ask the user to adjust the camera angle for a better view to achieve more accurate recognition results.

Third, the agents are often interested in recognizing specific texts on the screen and can mark the region of interest for the AI to process. This approach helps improve the processing speed of AI models and reduces unwanted, distracting outputs from the models.

Note that the above three aspects of human–AI collaboration overlap, e.g., the enhanced camera feed, can be utilized by both humans and AI to improve recognition capabilities. Since it is still an open problem, there may be other aspects of human–AI collaboration to explore in the future. For example, to train AI models specifically for digital text on LCD screens, we need volunteers to collect images of digital content from LCD screens or signage from various sources (e.g., the Internet, self-taken photos) under different conditions (e.g., image resolution, character size, brightness, blurriness) and annotate the location and content of the text in the images. The VizWiz [142] dataset has set one such precedent. This dataset contains over 31,000 visual questions originating from blind users who captured a picture using their smartphone and recorded a spoken question about it, together with 10 crowdsourced answers per visual question.

### 6.4. Emerging Problem 4: Recognizing Texts on Irregular Surfaces

Reading important information on irregular surfaces (e.g., non-orthogonal orientations, curved surfaces) is common in PVI’s lives, such as reading the instructions on medical bottles and checking the ingredients on packaged snacks or drink bottles. However, it is extremely challenging for agents to recognize text on irregular surfaces through the camera feed [43] due to the distorted text and unwanted light reflection, as listed in problem G2.(1) in Table 1. Therefore, we identify an emerging research problem of reading text on irregular surfaces that can be addressed by human–AI collaboration.

As far as only AI techniques are considered, scene text detection and recognition methods [135] could offer possible solutions to this problem based on the discussions in Problem 3. However, the weaknesses of pure AI solutions are similar to those in Problem 3. First, the state-of-the-art scene text recognition methods [136-138] still cannot perform satisfactorily on benchmark datasets. Second, existing text recognition methods [135] mostly read text on flat surfaces, and there are no methods specifically designed for recognizing text on irregular surfaces. When directly applying existing methods to reading text on irregular surfaces, the recognition accuracy would degrade further due to the text distortion and light reflection.

Without regard to human–AI collaboration, scene text recognition methods [135] read text by only relying on the trained AI models but not considering human inputs, while existing RSA services take no account of the potential applications of AI-based methods. Similar to Problem 3, we consider three main aspects of human–AI collaboration in recognizing text on irregular surfaces. *First,* the CV techniques can rectify the irregular content [143] and augment the video (e.g., deblurring the text [144]), and the agents recognize the text from the augmented video. *Second,* the agents can ask the user to move/rotate the object (e.g., medicine bottle) or change the camera angle to have a better view, and the AI models [135] can help recognize the text, especially the small characters. *Third,* the agents can select the region of interest on the irregular surfaces in the video for AI to process by either augmenting display or recognizing text. In addition, volunteers may be needed to collect images of text on different irregular surfaces (e.g., round bottles, packaged snacks) with various conditions (e.g., image resolution, character size, viewing angle) and annotate them for training customized AI models.

Despite similarities, there are three main differences between Problem 3 and Problem 4: (i) Problem 3 addresses the text recognition problem for luminous digital screens, but Problem 4 focuses on the text on non-luminous physical objects. (ii) The text in Problem 3 is on planar screens, but Problem 4 addresses the recognition on irregular (e.g., curved) surfaces. Thus, they require different customized AI models. (iii) The screens in Problem 3 are usually fixed, and the user can move the camera to get a better viewing angle. In contrast, the objects with text in Problem 4 are movable. For example, the user can rotate the medicine bottle and change the camera angle. That is, Problem 4 supports more interaction patterns than Problem 3.

### 6.5. Emerging Problem 5: Predicting the Trajectories of Out-of-Frame Pedestrians or Objects

**Figure 2.**
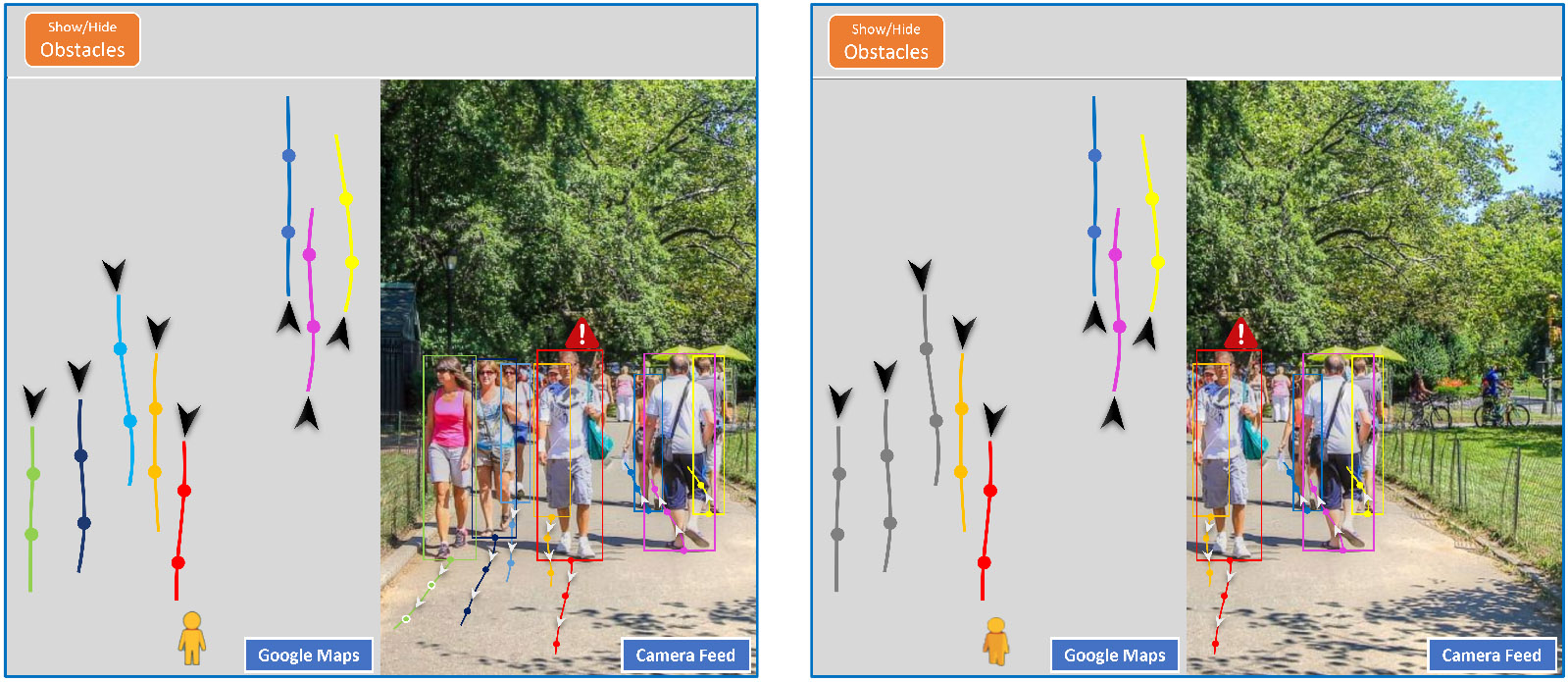
Our design prototype for predicting the trajectories of out-of-frame pedestrians. The top toolbar in each figure shows buttons to toggle the design feature on or off. The information on indoor maps and the camera feed is coordinated through colors. Rectangles represent pedestrian detection, lines on the ground are trajectory predictions, intervals between dots symbolize equal distance, arrows represent orientation, and alerts will pop up when collisions may occur. Trajectories of pedestrians turn gray when the pedestrians are out of the camera feed, as shown in Figure 2b.

In RSA services, the agents need to provide the environmental information in the user’s surroundings (e.g., obstacles and pedestrian dynamics) for safety when the user is in a crowded scene. The trajectory prediction of pedestrians or moving objects could assist the agent in providing timely instructions to avoid collision. According to our literature review, it is extremely difficult for RSA agents to track other pedestrians/objects [43,94] from the users’ camera feed and almost impossible to predict the trajectories of out-of-frame pedestrians or objects [8-11,13,42,94], as listed in problems G2.(3) and G2.(4) in Table 1. The main reasons are the narrow view of the camera and the difficulty in estimating the distance. Based on this observation, we pose an emerging research problem of predicting the trajectories of out-of-frame pedestrians or objects that can be addressed by human–AI collaboration, as illustrated in Figure 2.

Considering only AI solutions, we can adopt human trajectory prediction technology [96], which has been studied as a CV and robotics problem. Specifically, the motion pattern of pedestrians/objects can be learned by a data-driven behavior model (e.g., deep neural networks). Then, based on the observation from the past trajectories, the behavior model can predict the future trajectories of the observed pedestrians/objects. There are two types of problem settings, i.e., observed from either static surveillance cameras [145,146] or moving (handheld or vehicle-mounted) cameras [147,148]. For RSA application, we focus on predicting from handheld cameras. Existing trajectory prediction methods forecast the future pixel-wise locations of the pedestrians on the camera feed without considering the out-of-frame cases. The pixel-level prediction is also not useful for the agents to estimate the distance to avoid collision. Moreover, existing models are learned from the scene without PVI, but the motion patterns of pedestrians around PVI could be rather different.

In terms of human–AI collaboration, to the best of our knowledge, there is no work exploring the problem of pedestrian tracking and trajectory prediction under active camera controls. We consider three aspects of human–AI collaboration in predicting the trajectories of out-of-frame pedestrians. *First,* we need to develop user-centered trajectory prediction technologies. On the one hand, the behavior models need to be trained from a PVI-centered scene. On the other hand, the predicted trajectories should be projected to the real world where even the pedestrians cannot be observed from the camera feed. Based on such trajectory predictions, the agents can quickly plan the path and provide instructions to the user. *Second,* the agents may be only interested in the pedestrian dynamics toward the user’s destination. In this case, the agents can mark the region of interest for AI models to conduct prediction. Then, AI models will save some computational resources and also understand the interests of the agents. *Third,* in turn, AI models could suggest moving the camera towards a certain direction (e.g., left) to obtain more observations for better predictions. In this way, AI models can better reconstruct the scene for the agents to make navigational decisions for the user. This problem can be further extended in the human–AI collaboration setting. For example, AI could offer suggestions on the user’s walking directions with motion planning algorithms [149] based on the prediction results.

### 6.6. Emerging Problem 6: Expanding the Field-of-View of Live Camera Feed

In the RSA service, the sighted agent acquires information mainly from the live camera feed. However, prior work [9] found that the FOV of the camera in RSA was around 50°, much smaller than that of human vision, which is up to 180°. The narrow FOV of the live camera feed negatively affects the guiding performance of RSA agents [13,108]. Specifically, the main cause of problem G2, “obstacle and surrounding information acquirement and detection”, in Table 1 is due to the small FOV of the camera. For example, with the live camera feed, the agent is unable to project or estimate out-of-frame objects or obstacles from the user’s camera feed, i.e., problem G2.(1) in Table 1. Based on the significant impact of the camera FOV on RSA performance, we identify an emerging research problem of expanding the FOV of live camera feed that can be addressed by human–AI collaboration.

Although the problem of a narrow FOV is inherently limited by the specifications of smartphone cameras, it is possible to expand the FOV using computer vision or computational imaging techniques. We consider two possible solutions (i.e., fisheye lens, 3D mapping) and their human–AI collaboration opportunities.

**Figure 3.**
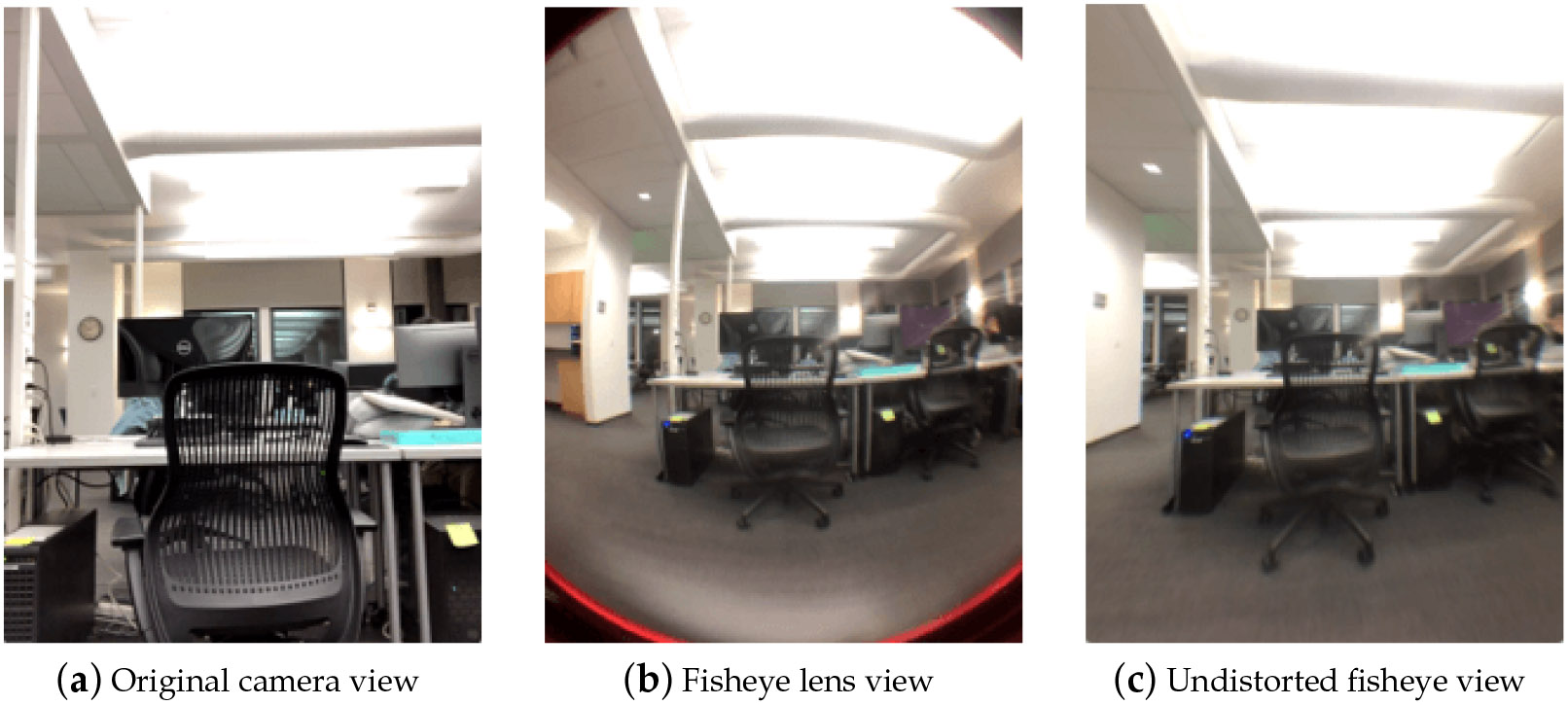
Expanding the field of view with fisheye lens. Here, we attached a fisheye lens to the rear-facing camera of an iPhone 8 Plus.

The first solution is attaching a fisheye lens to the smartphone camera, as shown in Figure 3. The fisheye lens distorts the straight lines of a perspective with a special mapping to create an extremely wide angle (e.g., 180°) of view. After attaching the fisheye lens, the appearance of the live camera feed will be “distorted”, i.e., convex non-rectilinear instead of rectilinear, as shown in Figure 3b. We can undistort the live camera feed to a regular rectilinear video using CV toolkits (e.g., OpenCV [150]) with the calibrated camera parameters. The undistorted fisheye view can obtain a larger FOV than the original camera view, as seen in Figure 3c. In terms of human–AI collaboration, there are two key issues for the RSA agent to decide: whether to use a fisheye lens and whether to apply the undistortion algorithm. (i) Despite the larger FOV, the camera view from the attached fisheye lens will have a reduced video resolution, compared with the original camera view. In this sense, the RSA agent needs to decide whether to use the fisheye lens for different tasks. For example, in a navigational task, the RSA agent would probably benefit from the fisheye lens to better avoid obstacles and get familiar with the surroundings. However, in a recognition task, the RSA agent may need to observe local details with high-resolution videos and not use the fisheye lens. (ii) The fisheye lens can provide a focus-plus-context display effect [151], which may be better than the undistorted view for the agents to complete tasks with a view focus. Additionally, the undistorted view is usually cropped to maintain a display without much blank area. However, the advantage of the undistorted view is obvious. It can provide a natural perspective view for the agent, especially beneficial for the distance estimation problem G2.(2) in Table 1. According to these factors, the RSA agent needs to decide in different tasks whether to apply undistortion.

The second solution is based on 3D mapping. We can build offline high-quality 3D maps for the target surroundings with necessary annotations. During online RSA, the 6-DOF camera pose in the 3D map can be obtained by CV algorithms. Given the camera pose, we can render an environmental image with any size of FOV from either the horizontal or vertical direction by changing the intrinsic parameters (e.g., focal length) of the virtual camera [99]. In this way, the agent can obtain structural information about the surroundings, which is especially useful for navigational tasks. In terms of human–AI collaboration, the agent needs to specify how much the view would be expanded, i.e., what the size of the field of the rendered view it would be. Because the central part of the view is most important, it is not the case that the larger view is better. During assistance, the agent may be more interested in one part than others on the live camera feed. Thus, another input from the agent could be which part should be extended.

The two solutions for expanding FOV of live camera feed have their own advantages. Attaching a fisheye lens is a simple solution but can only provide limited expansion. The solution with 3D mapping can expand the view to any size but may provide misleading information, because the current scene could be different from the 3D map, especially for dynamic objects. Additionally, the second solution can only be applied to mapped scenes. Both solutions need human inputs to achieve the optimal effect, which provides research opportunities for human–AI collaboration.

### 6.7. Emerging Problem 7: Stabilizing Live Camera Feeds for Task-Specific Needs

The quality of the live camera feed, especially the steadiness and clearness of the video, can greatly affect the RSA performance in hazard recognition [109,110], and thus, it is considered as one of the main factors determining the safety of blind users [10]. The problem of unstable camera feed is identified as G2.(5) in Table 1. Prior works [11,94] found that the camera should be located appropriately to maximize vision stability. Since there is no camera stabilizer for either handheld mobile devices or glasses in RSA service, the live camera feed is easily affected by the camera motion and becomes unstable. It is reported that agents are more likely to experience motion sickness when the users are not holding the camera (e.g., smartphone hanging around the user’s neck) [43]. Based on the significance of stabilizing live camera feeds, we regard it as an emerging research problem that can be addressed by human–AI collaboration.

From the perspective of AI techniques, video stabilization methods [100] could offer possible solutions to this problem. These methods usually first analyze the video frames to estimate the camera motion trajectory. For a shaky video, the camera motion is also oscillatory. Then, a low-pass filter is applied to smooth the camera motion. Based on the smoothed camera motion and the original shaky video, a new stabilized video can be synthesized. In this pipeline, the strength of stabilization does not have an objective criterion.

In terms of human–AI collaboration, the stabilization of the live camera feed needs the agent’s input on the strength of stabilization, which corresponds to the parameters of the low-pass filter. For various RSA tasks, the needed strengths of stabilization are different. For example, in a navigational task when the user is walking, the goal of stabilization is to reduce the video shaking and generate a pleasing video feed for the agent; in a text reading task when the user is trying to stay static, the stabilization should make the text display as still as possible. It is worth noting that the captured frame image can be blurry due to the camera shake. Based on the display effect of video stabilization, the agent can further adjust the strength of stabilization to achieve the optimal effect for a specific task. In addition, the study of Problem 7 (stabilizing live camera feeds) can also benefit Problem 3 (recognizing digital content) and Problem 4 (recognizing texts on irregular surfaces).

### 6.8. Emerging Problem 8: Reconstructing High-Resolution Live Video Feeds

As discussed in Problem 7, the quality of the live camera feeds can significantly affect the RSA performance. Besides the steadiness, the clearness is also an important aspect of the quality of the live camera feed, especially for recognition tasks. Limited by the configuration of the user’s smartphone camera and the transmission bandwidth [43], the resolution of the live camera feed showing to the agent is not always high enough to support recognizing small objects. The enhancement of the low-quality video feed is a major problem in RSA services, as listed in problem G4.(2) in Table 1. Therefore, we identify an emerging research problem of reconstructing high-resolution live video feeds that can be addressed by human–AI collaboration.

If only considering AI techniques, we can adopt video super-resolution methods [106]. Video super-resolution aims to reconstruct a high-resolution video from the corresponding low-resolution input. It is a classic CV problem but also challenging. The state-of-the-art video super-resolution methods [152-154] delicately design deep neural networks and train the models with large-scale labeled datasets. When applying these models to RSA services, a major bottleneck is the high complexity of the models and the limited computational resources for processing the full-size video.

To overcome this limitation, we can resort to a solution based on human–AI collaboration. In RSA services, the agent is usually interested in viewing the details of a certain part of the live video feed. For example, in the task of reading signages and texts, the agent only cares about the area with signages and texts on the video. Therefore, the video super-resolution model can be run only on the portion of the video specified by the agent. In this way, we can have enough computational resources to only process the relevant part. Similar to Problem 7, the study of Problem 8 (reconstructing high-resolution live video) can directly benefit Problem 3 (recognizing digital content) and Problem 4 (recognizing texts on irregular surfaces).

### 6.9. Emerging Problem 9: Relighting and Removing Unwanted Artifacts on Live Video

We have discussed the shaky and low-resolution issues affecting the quality of the live camera feed in Problem 7 and Problem 8, respectively. There are other important aspects of the quality of the live camera feed that can greatly impact the RSA performance. The first issue is the poor lighting condition, either too dark or too bright. As listed in problem G4.(2) in Table 1, low ambient light conditions at night cause the poor quality of the video feed. The second issue is due to the unwanted artifacts on the live video, including unwanted lights (e.g., reflected light, flare or glare) and unwanted contaminants (e.g., dust, dirt, and moisture) on the camera lens. These issues also belong to the external issues of problem G4 in Table 1 as major RSA challenges. Therefore, we identify an emerging research problem of changing the lighting condition (or simply relighting) and removing unwanted artifacts on live video that can be addressed by human–AI collaboration.

For the issue of poor lighting conditions, there exist extensive AI-based methods in the literature on illumination estimation and relighting [107] in computer vision and computer graphics. Basically, the original lighting of the scene in the video can be recorded as high dynamic range (HDR) light probes [155]; then the lighting of the original video can be accordingly adjusted to be pleasant to view. These methods cannot be directly applied to RSA services, because the scene after relighting may look quite different from what other pedestrians see and may mislead the agent to make wrong judgments. This issue can be addressed in a human–AI collaboration framework, where the agent can define the type of the scene (e.g., indoor or outdoor) and specify the strength of the relighting effect.

For the issue of unwanted artifacts, there are also AI-based methods for removing unwanted lights [156] and unwanted contaminants [157] in the CV literature. These methods train deep neural models with synthesized datasets to detect unwanted lights (e.g., scattering flare, reflective flare) or unwanted contaminants and restore the unwanted parts with view synthesis. When applying to the RSA services, AI-based methods can be further improved with the human–AI collaboration framework. On the one hand, the agent can ask the user to act to reduce the influence of the unwanted artifacts (e.g., move the camera direction to avoid flare, or wipe the lens to clean some contaminant). On the other hand, the agent could identify the unwanted artifacts on the live video feed and mark the area for the AI algorithm to conduct restoration. The AI-detected contaminant can be approved or denied by the agent. In this way, the removal results would be better than AI-only methods.

In addition, the study of Problem 9 can help address Problem 4 (recognizing texts on irregular surfaces), where light reflection or refraction is one of the main challenges in recognizing texts on irregular surfaces.

### 6.10. Emerging Problem 10: Describing Hierarchical Information of Live Camera Feeds

In RSA services, the agents need to deliver the information from the live camera feed to the users and interact with them constantly. Previous works [1,101] found the agents having difficulty in providing various required information (e.g., direction, obstacle, and surroundings) in a timely manner. It makes it even more challenging that the agents need to prioritize the information, which requires understanding and communicating with users. Additionally, the agents could stress out if the users move faster than they could describe the environment [1]. These suggest that the requirement of describing a large volume of information to meet the user’s needs is a major challenge in RSA services, as listed in problem group G3 in Table 1. Based on this observation, we introduce an emerging research problem of describing hierarchical information of live camera feeds that can be addressed by human–AI collaboration.

From the perspective of AI solutions, there are some CV methods of image captioning [158,159] applied for describing the content of an image to visually impaired people with natural language. These methods [159] only show the possibility of using image captioning techniques for PVI, but are not used for practical applications such as describing information from the live camera feed in RSA services. There are also a few CV systems that answer visually impaired people’s questions about an image using visual question answering (VQA) techniques [160,161]. These systems are only academic prototypes tested in lab settings. The state-of-the-art model [161] only achieves the performance of IoU similarity at less than 30% on benchmark datasets, which cannot be compared with human performance. Thus, these VQA systems are far from interacting with the user in RSA.

Since both the agent and the AI methods face great challenges in delivering information to the user in RSA services, we can resort to human–AI collaboration solutions to address the challenge. Considering that humans perform much better than existing VQA techniques, the agent should take the main responsibility for communicating with the user. Meanwhile, we can use object detection and video captioning methods [105] to help the agent organize the information in a hierarchical structure with priorities for the items. For example, the items related to safety (e.g., detected obstacles) should be prioritized. The VQA model can answer the user’s simple questions such as “what is in front of me?” When the agent needs to start another parallel task (e.g., browsing the map), the video caption and VQA models can assist the user by describing the scene and answering simple questions, respectively. With the AI assistant, the agent will feel less stress in describing the information from live video feeds.

## 7. Integrating RSA with Large Language Models (LLMs)

### 7.1. AI-Powered Visual Assistance and LLMs

AI-powered visual assistance, unlike RSA, which depends on human agents, does not rely on human intervention but instead assists PVI through AI agents. The progress in deep learning models for CV and NLP technologies has notably bolstered the functionalities of AI-powered visual assistance systems. These systems utilize images captured by individuals with PVI to detect objects or text within the scene, and they can also provide responses to inquiries about the image contents. As an example, Ahmetovic et al. [162] created a mobile application that employs deep learning models to assist individuals with PVI in capturing images and identifying objects. Hong et al. [163] developed an iOS application that empowers PVI to collect training images for personalized object recognition. Morrison et al. [164] crafted an application aimed at instructing AI to recognize personalized items, thereby assisting PVI in locating their personal belongings. Gonzalez et al. [165] introduced a scene description application leveraging Microsoft’s Azure AI Vision image description API. Moreover, PVI frequently utilize commercial AI-powered applications such as Microsoft’s Seeing AI [166] to identify objects via device cameras.

In recent times, large language models (LLMs), particularly multimodal large language models (MLLMs), as discussed in Yin et al.’s survey [15], and large vision language models (LVLMs) like GPT-4V [16], have demonstrated impressive capabilities in visual comprehension and reasoning. Consequently, researchers have initiated investigations into how LLMs can support PVI. Zhao et al. [167] explored the viability of harnessing cutting-edge LLMs to assist PVI and established a relevant benchmark. Similarly, Yang et al. [168] employed LLMs to craft an assisting system, VIAssist, aimed at addressing PVI’s queries regarding captured images, including evaluating image quality, and suggesting potential retakes.

In the commercial sphere, BeMyEyes [6] partnered with OpenAI to launch the BeMyAI feature [17], leveraging the capabilities of GPT-4 [16] with the goal of substituting human volunteers. Users have utilized BeMyAI to access a wide array of information [17], spanning from product details and usage instructions to culinary guidance like popcorn recipes, appliance operation tips, wardrobe organization strategies, device setup assistance, reading materials such as comics and books, configuring streaming devices, locating misplaced items, decoding memes across various social media platforms, obtaining detailed descriptions of artworks and event photos, accessing transportation schedules, perusing restaurant menus and receipts, translating text, receiving academic support, and identifying beauty products while checking makeup. Recently, Xie et al. [169] conducted an exploratory study with 14 visually impaired participants to explore the use of LLM-based assistance tools like BeMyAI in their daily lives. The study revealed that BeMyAI significantly enhances visual interpretations and offers thoughtful insights on visual content, enabling novel use cases and practices that were not possible with earlier AI models.

### 7.2. Opportunities for human–AI Collaboration in RSA with LLMs

#### 7.2.1. Human Agent Supporting LLM-Based AI

While LLMs have experienced remarkable advancement in recent years, their accuracy and reliability remain inadequate for effectively aiding PVI in accomplishing diverse complex life tasks. Current AI-powered assistance systems relying on LLMs are primarily confined to tasks like scene description and VQA [17]. However, for intricate endeavors such as navigating complex environments that necessitate comprehension of 3D space, the current LLM-based assistance systems still struggle. Thus, the involvement of human agents is essential to successfully tackle these multifaceted tasks.

**Figure 4.**
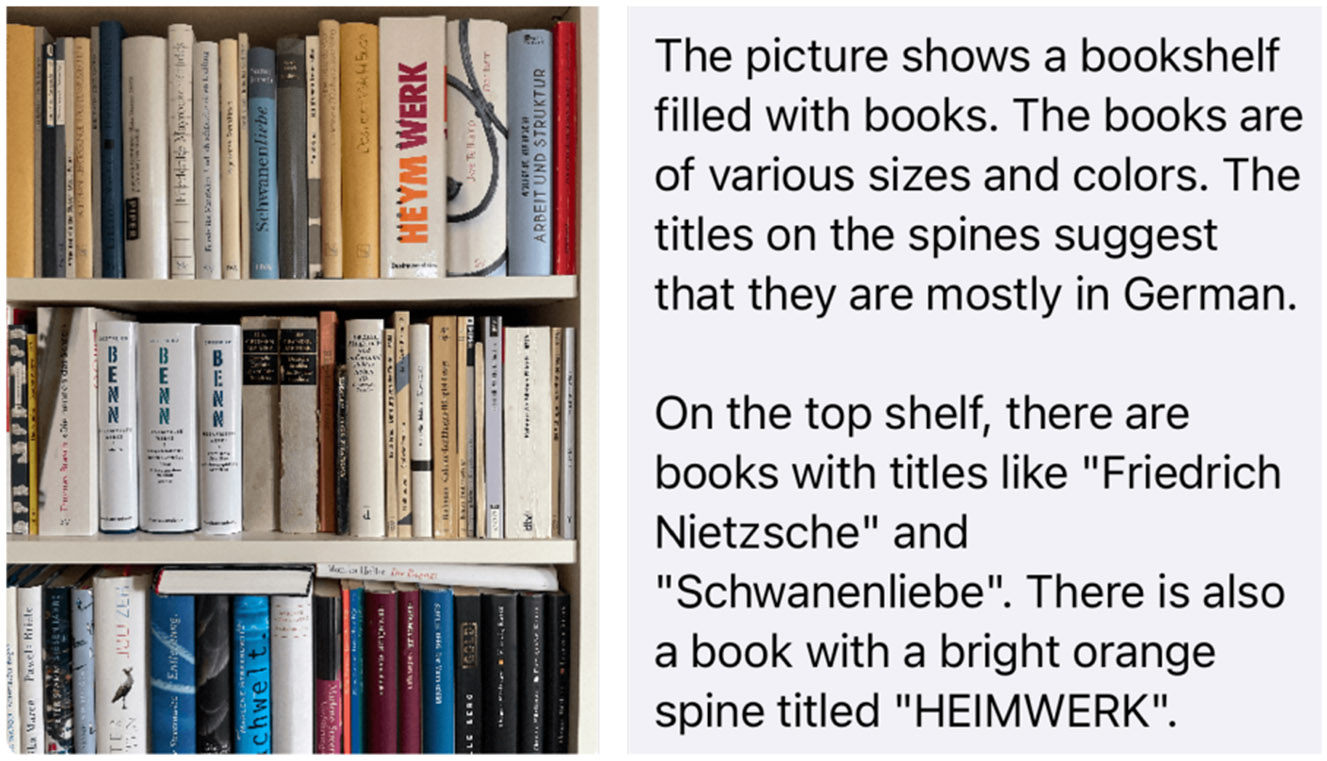
Example output of BeMyAI [17]. The German book title *HEYMWERK* was erroneously identified as *HEIMWERK* (image source: [170]).

Even for simple recognition tasks, LLMs are not flawless, let alone handling complex ones. Bendel [170] detailed an encounter with BeMyAI and found some errors and limitations, including occasional misclassifications and inaccuracies in object recognition, such as mistaking wall paneling for cupboards or large windows for doors. In some cases, the app incorrectly identifies or describes objects, like a bear on a shelf that was not there, or misinterprets text, as seen in the misreading of a book title. As shown in Figure 4, the German book title *HEYMWERK* was mistakenly interpreted as *HEIMWERK*. Human agents can readily rectify such recognition errors with ease.

#### 7.2.2. LLM-Based AI Supporting Human Agent

In Section 6, we have pinpointed 10 emerging problems where AI supporting RSA can be applied, particularly in leveraging CV to aid human agents in terms of scene information acquisition and live video enhancement. Beyond the collaborative prospects outlined in Section 6, the enhanced image comprehension and reasoning abilities of LLMs offer significant potential for bolstering the perceptual and intellectual support provided to human agents.

Regarding perceptual support, given the limitations of human cognitive capacity, human agents often struggle to swiftly identify target objects amid complex images featuring numerous elements. Leveraging LLM-based AI can expedite target localization within images, thanks to its robust computational capabilities. In [167], Zhao et al. presented an example of an input image featuring numerous items arranged on multiple shelves within a grocery store. LLMs can swiftly offer location cues for the target item (e.g., mustard) within such complex scenes.

In terms of intellectual support, LLMs draw from vast amounts of Internet data for training, surpassing the knowledge scope of human agents by a considerable margin. A clear illustration of this is when a task involves specialized or unfamiliar information; human agents, lacking exposure to such data, are unable to offer meaningful assistance to PVI. For instance, if confronted with an unfamiliar trademark, human agents may struggle to provide product details to PVI or even find relevant information through search engines like Google. In contrast, LLMs-based AI likely possesses this information and can furnish it to human agents. Additionally, LLM-based AI inherently possesses multilingual capabilities, enabling communication with individuals speaking diverse languages, a feat beyond the reach of ordinary human agents.

### 7.3. Human-Centered AI: Future of Visual Prosthetics

Based on the above discussion, both human agents and LLM-based AI have distinct impacts on visual prosthetics. We posit that human-centric AI represents the fundamental iteration of visual prosthetics in the future. With AI advancing in capability, the nature of RSA will undergo further evolution. Consequently, the dynamics between humans and AI within the realm of visual prosthetics will also undergo ongoing transformation.

According to a previous study [165], PVI participants tend to opt for AI usage solely for uncomplicated tasks to reduce social burdens. In this research, PVI participants employed AI-powered applications for tasks they deemed trivial or unworthy of bothering human assistants. For example, they utilized AI to distinguish between sunglasses and prescription glasses, as they preferred not to inconvenience others with such inquiries. They perceived AI as a means to spare their social circle from the weight of requesting answers to seemingly minor questions.

A previous study [165] also found that PVI opted for AI to access impartial information. PVI participants regarded AI as an impartial information outlet, especially in visual information disputes. For instance, they employed AI as a mediator to resolve disagreements, such as determining the accuracy of the view from an airplane window. They relied on AI to offer unbiased judgments in these scenarios.

**Figure 5.**
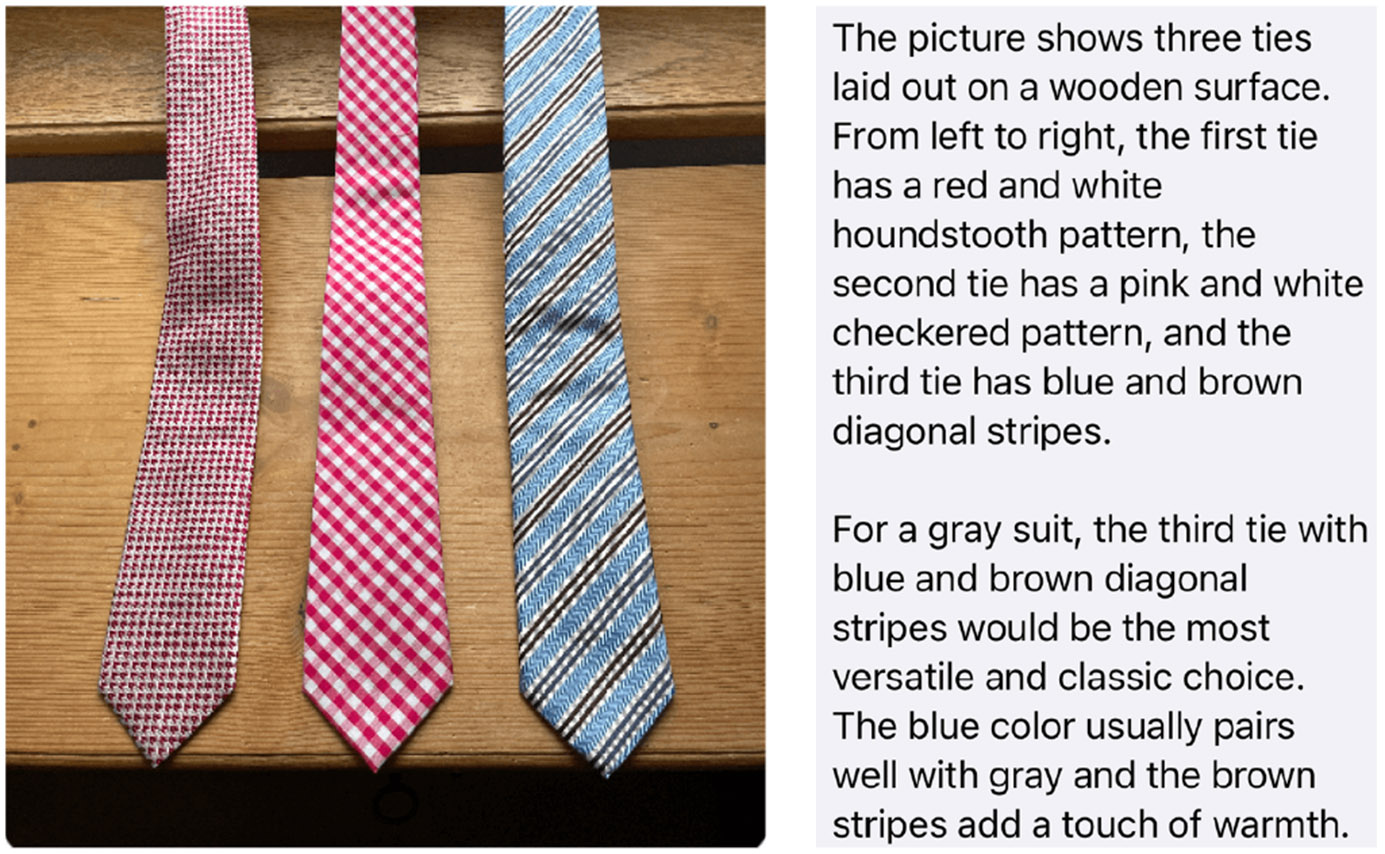
BeMyAI can offer subjective tie and suit pairing suggestions, accompanied by explanations (image source: [170]).

Our discourse on human–AI collaboration can draw inspiration from research on human–human collaboration. Xie et al. [171] conducted a study on RSA involving paired volunteers. They discovered that paired volunteers were more effective in tackling open-ended tasks, particularly those demanding subjective opinions. In traditional RSA, volunteers typically offer objective descriptions. However, when it comes to subjective opinions, human–AI collaboration holds distinct advantages, as AI models like LLMs have assimilated broad perspectives and aesthetic sensibilities from vast online datasets. As depicted in Figure 5, in scenarios like choosing a tie to match a gray suit, BeMyAI can furnish subjective suggestions grounded in clothing matching expertise, accompanied by rationales [170].

Moreover, paired volunteers facilitate PVI engagement in more intriguing activities, such as appreciating art. As generative AI progresses, human–AI collaboration stands poised to further enrich the life experiences of PVI. For instance, leveraging AI tools for artistic endeavors presents a promising avenue. Presently, sighted individuals can utilize generative AI tools to generate images from text inputs (e.g., Midjourney [172], DALL·E [173]), or craft videos (e.g., Sora [174]). Through human–AI collaboration, PVI participants are poised to delight in the creative possibilities offered by AI tools. This nascent research direction warrants further exploration.

Furthermore, a potential future trajectory involves the customization of personalized AI for individual users, diverging from the use of generalized LLM models. This personalization can be attained through ongoing user–AI interactions. For instance, a personalized AI would become acquainted with a PVI user’s language preferences and lifestyle routines and even recognize the user’s personal belongings. In contrast with randomly selected human agents in crowdsourcing scenarios, personalized AI holds greater promise in catering to the specific needs of PVI. To enable personalized AI, user profiling [175,176] may enhance the training of these AI models effectively. For example, profiling users’ height and walking stride length [177] could improve the customization of AI models and RSA services, providing better navigational support.

LLM-based AI is poised to revolutionize the landscape of visual prosthetics. The future evolution of visual prosthetics will be influenced by numerous factors, including the accuracy and reliability of AI, autonomy requirements, social considerations, privacy concerns, and more. Whether utilizing human-assisted or AI-driven support, visual prosthetics will grapple with common ethical and social dilemmas. Take privacy [178,179], for instance: in current RSA setups, human agents may inadvertently intrude on the privacy of PVI through camera interactions, while AI agents may glean insights into users’ personalities, posing potential privacy risks. Given the uncertain trajectory of visual prosthetics, these ethical and social quandaries remain open for exploration.

## 8. Conclusions

In this paper, we synthesize an exhaustive list of challenges in agent-user interaction within RSA services through a comprehensive literature review and a study involving 12 visually impaired RSA users. We then analyze the CV problems related to these challenges, demonstrating that some cannot be resolved using current off-the-shelf CV techniques due to the complexity of the underlying issues. We propose that these challenges can be addressed through collaboration between RSA agents and CV systems. To this end, we formulate 10 emerging human–AI collaboration problems in RSA. Additionally, we explore potential approaches for integrating LLMs into RSA and discuss the future prospects of human–AI collaboration in the LLM era. We summarize the emerging problems and proposed human–AI collaboration approaches in Table 3, along with three common human–AI collaboration strategies for different emerging problems.

Current commercial RSA services, such as BeMyEyes [6] and Aira [7], rely solely on human agents or volunteers to provide assistance, while CV-based assistive technologies [165] depend entirely on AI to interpret the scene. Our study offers an intermediary solution: human–AI collaboration, which aims to more effectively assist PVI in completing various tasks. This collaborative approach, especially in the LLM era, is poised to become increasingly important and represents a significant direction for future visual prosthetics.

## Figures and Tables

**Table 1.** A list of challenges in RSA service and related CV problems, presented in four groups (G1, G2, G3, and G4).

	Challenges	CV Problems
G1.	Localization and Orientation	
(1)	Scarcity of indoor map [1,13,19,85]	Visual SLAM [86]
(2)	Unable to localize the user in the map in real time [1,10,42,85]	Camera localization [87]
(3)	Difficulty in orienting the user in his or her current surroundings [9,19,42]	Camera localization [87]
(4)	Lack of landmarks or annotations on the map [1,62]	Landmark recognition [88]
(5)	Outdated landmarks on the map [1,62]	Landmark recognition [88]
(6)	Unable to change scale or resolution in indoor maps [1]	
(7)	Last-few-meters navigation (e.g., guiding the user to the final destination) [26,85]	Visual navigation [89]
G2.	Obstacle and Surrounding Information Acquirement and Detection	
(1)	Difficulty in reading signages and texts in the user’s camera feed [43]	Scene text recognition [90]
(2)	Difficulty in estimating the depth from the user’s camera feed and in conveying distance information [13]	Depth estimation [91]; scale estimation [92,93]
(3)	Difficulty in detecting and tracking moving objects (e.g., cars and pedestrians) [43,94]	Multiple object tracking [95]; human trajectory prediction [96,97]
(4)	Unable to project or estimate out-of-frame objects, people, or obstacles from the user’s camera feed [8-11,13,42,94]	Human trajectory prediction [96,97]; FOV extrapolation [98,99]
(5)	Motion sickness due to unstable camera feed [43]	Video stabilization [100]
G3.	Delivering Information and Understanding User-Specific Situation	
(1)	Difficulty in proving various information (direction, obstacle, and surroundings) in a timely manner [1,101]	Object detection [102]; visual search [103,104]
(2)	Adjusting the pace and level of detail in description provision through communication [1,43]	Video captioning [105]
(3)	Cognitive overload	
G4.	Network and External Issues	
(1)	Losing connection and low quality of video feed [1,13,42,43,45,94]	Online visual SLAM [86]
(2)	Poor quality of the video feed	Video super-resolution [106]; video relighting [107]

**Table 2.** All 15 scenarios were reported by all RSA users. Scenarios with * occurred more frequently than others. Additionally, participants perceived these as more challenging than others.

Outdoor Scenarios	Indoor Scenarios
1. Going to mailbox	1. Finding trash cans or vending machines
2. Taking a walk around a familiar area (e.g., park, campus)	2. Finding architectural features (e.g., stairs, elevators, doors, exits, or washrooms)
3. Walking to the closest coffee shop	3. Finding a point of interest in indoor navigation (e.g., a room number, an office)
4. Finding the bus stop	4*. Navigating malls, hotels, conference venues, or similarly large establishments
5*. Crossing noisy intersections without veering	5. Finding the correct train platform
6. Calling a ride share and going to the pick-up location	6. Navigating an airport (e.g., security to gate, gate to gate, or gate to baggage claim)
7*. Navigating from a parking lot or drop-off point to the interior of a business	7. Finding an empty seat in theaters or an empty table in restaurants
8*. Navigating through parking lots or construction sites	

**Table 3.** A summary of emerging human–AI collaboration problems in RSA.

	Emerging Problems in RSA	Current Status of Research	Proposed Human-AI Collaboration
1	Motivated by the identified challenges		
(1)	Making object detection and obstacle avoidance algorithms blind aware	•Existing object detection algorithms [119,120] are not blind aware.	•Human annotation of blind-aware objects for training and updating AI models.
(2)	Localizing users under poor networks	•No prior work for large delays or breakdowns of video transmissions [13,94,111]	•Using audio and camera pose in 3D maps •Interactive verification of camera pose
(3)	Recognizing digital content on digital displays	•No recognition systems for digital texts •OCR [135] suffers from domain shift [140]	•AI-guided adjustment of camera view •Manual selection of AI recognition region
(4)	Recognizing texts on irregular surfaces	•No OCR systems for irregular surfaces •[135-138] Read text on flat surfaces	•AI-based rectification for human •AI-guided movement/rotation of objects
(5)	Predicting the trajectories of out-of-frame pedestrians or objects	•No such prediction systems •Existing models [147,148] only predict in-frame objects in pixels	•User-centered out-of-frame prediction •Agents mark the directions of interests •AI-guided camera movements
(6)	Expanding the field-of-view of live camera feed	•No prior work for real-time FOV expansion	•Task-specific use of fisheye lens •Human-customized view rendering
(7)	Stabilizing live camera feeds for task-specific needs	•Existing video stabilization methods [100] are developed for general purposes	•Task-oriented and adjustable video stabilization based on human inputs
(8)	Reconstructing high-resolution live video feeds	•Existing models [152-154] are limited by computational resources for live videos.	•Customized video super-resolution on certain parts based on human inputs
(9)	Relighting and removing unwanted artifacts on live video	•Existing models [107,156,157] are developed for general purposes (e.g., HDR [155])	•Human-guided custom relighting •Interactive artifact detection and removal
(10)	Describing hierarchical information of live camera feeds	•Captioning tools [158,159] are not for PVI •VQA for PVI [160,161] performs poorly	•AI helps agents organize information •Joint assistance by agents and AI
	*Common human–AI collaboration strategies for different emerging problems:* •AI-guided adjustment of camera views •Human-designated region for AI processing •Task-specific AI driven by human inputs		
2	Integrating RSA with LLMs		
(1)	Human agents enhancing LLM-based AI	•No prior work	•Human leading AI in intricate tasks •Human verifying AI for simple tasks
(2)	LLM-based AI supporting human agents	•No prior work	•Accelerating target localization with AI •AI-driven specialized knowledge support

## Abbreviations

The following abbreviations are used in this manuscript:

RSA: remote sighted assistance
VI: visual impairments
PVI: people with visual impairments
LLM: large language model
CV: computer vision
AI: artificial intelligence
AR: augmented reality
SLAM: simultaneous localization and mapping
DOF: degrees of freedom
LiDAR: light detection and ranging
OCR: optical character recognition
IMU: inertial measurement unit
FOV: field of view
O&M: orientation and mobility
LCD: liquid crystal display
HDR: high dynamic range
VQA: visual question answering
